# Psychological Characteristics and Quality of Life of Patients with Upper and Lower Functional Gastrointestinal Disorders

**DOI:** 10.1192/j.eurpsy.2023.865

**Published:** 2023-07-19

**Authors:** S.-Y. Lee, H.-J. Lee

**Affiliations:** 1 psychiatry, Wonkwang Univ. Hospital; 2 Public Health, Graduate School, Wonkwang University, Iksan, Korea, Republic Of

## Abstract

**Introduction:**

According to the psychodynamic hypothesis in FGIDs, as well, UGIDs such as functional heartburn (FH) and functional dyspepsia (FD) are often a consequence of receiving inadequate nutrition from one’s mother during childhood, which leads to a failure to adapt to eating. Meanwhile, lower GI disorders such as IBS and functional constipation are generally accompanied by avoidant defense mechanisms and obsessive compulsive disorder.

**Objectives:**

This study aimed to identify the differences in the psychological characteristics of the anatomical location of functional gastrointestinal disorders (FGIDs) and the factors that influence the quality of life (QOL).

**Methods:**

Altogether, 233 patients who were diagnosed with FGIDs were classified into the upper gastrointestinal disorder (UGID; n=175) group and the lower gastrointestinal disorder group (LGID; n=58). Psychological characteristics were identified using the Korean version of the Beck Depression Inventory 2^nd^ ed.; Korean version of the Beck Anxiety Inventory; Korean version of Childhood Trauma Questionnaire; Multi-dimensional Scale of Perceived Social Support; Korean version of Type-D Personality Scale-14; and Korean version of the Connor–Davidson Resilience Scale. QOL was evaluated using the World Health Organization Quality of Life - Brief Version.

**Results:**

The UGID group demonstrated higher scores in ‘emotional’ than the LGID group. (t=-3.031, p<.01) A significant difference was observed between groups in ‘significant others’. (t=2.254, p<.05) Significant differences were observed between the groups in hardiness (t=2.259, p<.05) and persistence (t=2.526, p<.05), while the LGID group demonstrated significantly lower scores than the UGID group in ‘negative affectivity’. (t=-1.997, p<.05) Additionally, the LGID group demonstrated lower QOL than the UGID group. (t=2.615, p<.05) The stepwise regression analysis on OQL involved depression, resilience, social support, and childhood trauma, which accounted for 48.4% of the total quality of life explanatory variance

**Conclusions:**

Psychological characteristics and QOL significantly differed when FGIDs were classified according to anatomical location. Thus, psychological interventions customized for each type of FGIDs may be necessary for effective treatment.

**Disclosure of Interest:**

None Declared

